# Psychosocial Work Factors and Musculoskeletal Pain: A Cross-Sectional Study among Swedish Flight Baggage Handlers

**DOI:** 10.1155/2015/798042

**Published:** 2015-10-19

**Authors:** Eva L. Bergsten, S. E. Mathiassen, E. Vingård

**Affiliations:** ^1^Centre for Musculoskeletal Research, Department of Occupational and Public Health Sciences, University of Gävle, 801 76 Gävle, Sweden; ^2^Occupational and Environmental Medicine, Department of Medical Sciences, Uppsala University, 751 85 Uppsala, Sweden

## Abstract

*Objective*. Flight baggage handlers sort and load luggage to airplanes. This study aimed at investigating associations between psychosocial exposures and low back and shoulder musculoskeletal disorders (MSDs) among Swedish flight baggage handlers. *Methods*. A questionnaire addressing MSDs (Standardized Nordic Questionnaire) and psychosocial factors (Copenhagen Psychosocial Questionnaire, COPSOQ) was answered by 525 baggage handlers in six Swedish airports. *Results*. Low back (LBP) and shoulder pain (SP) were reported by 70% and 60%, respectively. Pain was reported to interfere with work (PIW) by 30% (low back) and 18% (shoulders), and intense pain (PINT) occurred in 34% and 28% of the population. *Quality of leadership* was the most dissatisfying psychosocial factor, while the most positive was *social community at work*. Low ratings in the combined domain *Work organization and job content* were significantly associated with PIW in both low back and shoulders (Adjusted Hazard Ratios 3.65 (95% CI 1.67–7.99) and 2.68 (1.09–6.61)) while lower ratings in the domain *Interpersonal relations and leadership* were associated with PIW LBP (HR 2.18 (1.06–4.49)) and PINT LBP and SP (HRs 1.95 (1.05–3.65) and 2.11 (1.08–4.12)). *Conclusion*. Severity of pain among flight baggage handlers was associated with psychosocial factors at work, suggesting that they may be a relevant target for intervention in this occupation.

## 1. Background

Worldwide, more than 2000 airlines operated more than 23 000 aircrafts in 2006 [1]. These aircrafts made more than 28 million scheduled departures, carrying more than 2 billion passengers. A substantial proportion of these passengers would be expected to bring baggage that is checked in and thus handled by flight baggage handlers at the airports of departure and arrival. To a major extent, flight baggage handling services are similar in all larger airports, and so flight baggage handlers perform similar tasks all over the world.

Workers handling flight baggage are typically engaged in manual tasks like sorting, loading and unloading baggage, mail and flight cargo to the airplanes, and so-called airport ramp service work. The ramp is the area around the aircraft. In Swedish airports, bags checked in by passengers are placed on a conveyor belt, which transports the bags to a sorting area. In the sorting area, baggage handlers place the bags on a cart or in a Unit Load Device (ULD; i.e., a container that can be loaded on the aircraft), which is eventually transported by a truck to the ramp. There, the bags are loaded into the aircraft baggage compartment piece by piece, or in one operation for ULDs. Unloading an aircraft runs in reverse: bags are transferred from the aircraft compartment to the carts and transported by trucks to the arrival conveyor belt in the sorting area, which transports the bags to the arrival hall. Sometimes, baggage handlers engage also in communicating with air traffic controllers directing air traffic on the ground or engage in towing the aircrafts to and from gates with a pushback vehicle, and they may also serve aircrafts with auxiliary power units, brakes, and light.

Thus, flight baggage handling mainly consists in transferring baggage between carts and aircraft compartments, pushing and pulling loaded trailers, and stowing bags and freight, often while kneeling or squatting in confined compartments. Thus, baggage handling is associated with several physical factors suspected to increase risks for musculoskeletal disorders (MSDs), such as heavy manual materials handling, frequent lifting, awkward body postures, and pushing and pulling [2–7].

Scientific reports devoted to the baggage handling occupation are rare. One study concluded that the lower back was exposed to considerable loads when bags of different weights and destination height were handled in a kneeling posture in the compartment [8]. Two other studies investigated opinions about suspected causes of MSDs and effective prevention among baggage handlers and safety professionals [9, 10]. One of these studies reported a high prevalence of MSDs in back, knees, and shoulders among the baggage handlers [9].

None of the cited studies on risk factors and disorders among baggage handlers consider the possible role of the psychosocial work environment in causing or aggravating MSDs. It is suggested that psychosocial factors at work are associated with risks of developing disorders in the lower back, neck/shoulder region, and upper extremities [10, 11]. Evidence for poor social support as a risk factor for low back pain (LBP) was claimed in one review [11] and in a cohort study [12]. Lack of social support was also reported to be a risk factor for musculoskeletal morbidity, sickness absence, restricted activity, and not returning to work [13]. High emotional demands, low influence, and pronounced role conflicts at work have also been suggested to predict LBP [14]. The influence of psychosocial factors has been explained in the context of a biopsychosocial model [15], which emphasizes both mechanical and physiological processes in the generation and maintenance of pain, as well as the importance of psychological and social conditions for the response to pain and the disability developed by a particular individual.

Motivated by the lack of literature on psychosocial factors and their association with back and shoulder MSDs among flight baggage handlers, the aims of this study were as follows:

1. To conduct a nationwide Swedish survey of musculoskeletal disorders and psychosocial factors in the flight baggage handling occupation.
2. To determine the extent to which these psychosocial factors are associated with pain intensity and with pain interfering with work. We hypothesized less favourable psychosocial conditions to be associated with an increased likelihood of experiencing pain among the flight baggage handlers, as documented in several other occupational settings.

This study was part of a two-year work environment project among flight baggage handlers in Sweden, conducted during 2010–2012 under the auspices of the Vocational Training and Working Environment Council (TYA), a council formed by the Swedish aviation industry employers' association and the transportation worker union. TYA covers about 60% of all Swedish flight baggage handlers. The main goal of the overall project was to document physical and psychosocial work environment conditions, as a basis for developing interventions to improve health.

## 2. Methods

### 2.1. Airports and Baggage Handlers

Sweden has 41 airports with a total of about 1 400 baggage handlers employed for ramp service, either by a handling company or, at smaller airports, directly by the airport. All six handling companies affiliated to TYA in the three largest public Swedish airports (Stockholm-Arlanda, Göteborg-Landvetter, Malmö-Sturup) agreed to participate in the study; these companies are responsible for 75% of the yearly traffic at the three airports. In addition, all handlers working in three small private airports (Arvidsjaur, Småland and Skavsta) were approached. Baggage handlers working less than half-time were excluded, as well as handlers on vacation, parental leave, or sick leave by the time of data collection (December 2010 to April 2011). Altogether, 806 of the about 1400 baggage handlers working in Sweden were eligible for the study, which was approved by the regional Ethical Review Board in Uppsala.

### 2.2. Procedure

All companies and airports were visited by a member of the research team for a sufficient period of time to ensure that all handlers could be approached in person and informed about the study. The researchers handed out a questionnaire to each handler, which was, by most respondents, answered in about 25–30 minutes during working hours. The member of the research team collected questionnaires at that same occasion, but the handler also had the choice of sending the questionnaire later on to the researchers in a sealed envelope. Handlers who did not submit their questionnaires in the first place were approached again, in person, by member(s) of the research team. Completion and submission of the questionnaire were enthusiastically encouraged by local team managers and safety officers, but telephone numbers or addresses were not available for reminders.

### 2.3. Questionnaire

The questionnaire contained questions in six different areas: demographic factors, psychosocial factors, physical workload in different tasks, musculoskeletal disorders, general health, and fatigue.

### 2.4. Demographic Factors

The demographic factors included age, gender, height, weight, and years of experience as a baggage handler.

### 2.5. Psychosocial Factors

Psychosocial factors at work were assessed using the medium-length Copenhagen Psychosocial Questionnaire (COPSOQ) [16] in its latest edition, COPSOQ II [17]. In the present study, 13 scales with a total of 42 questions were used. The scales represent two main domains, that is,* Work organization and job content* (five scales) and* Interpersonal relations and leadership* (eight scales). Questions in six scales (*influence at work*,* variation*,* commitment to the workplace*,* social support from colleagues*,* social support from supervisors*, and* social community at work*) were answered using a five-step response ranging from “always” to “never/hardly ever.” In seven scales (*possibilities for development*,* meaning of work*,* predictability*,* recognition*,* role clarity*,* role conflicts*, and* quality of leadership*) questions were answered in five steps from “to a large extent” to “to a very small extent.” For all questions, the answer was transformed into a number between 0 and 100 (i.e., 0, 25, 50, 75, and 100) for the five response steps, and an overall scale score was computed as the mean score across questions contained in each of the 13 scales. A higher score indicated a more positive work environment, except for* role conflict* where a high score indicates more conflicts. For reasons of comparability questions included in the different scales were copied in their original form from the second version of the COPSOQ questionnaire, as reported by Pejtersen et al. 2010 [17] (see the appendix), and the procedure for calculating scale scores was also adopted from Pejtersen et al.

### 2.6. Physical Work Load

The perceived physical work load was rated for low back and shoulder separately in a number of tasks reported by the handlers to occur frequently in the job, using the question “how do you perceive the physical load in task xx” with answers on a six-grade scale from “not at all” to “to a large extent.” For 31 baggage handlers, the occurrences of these tasks were determined from video recordings collected by a member of the research team for half a work shift. Across these 31 baggage handlers, the mean time proportions of the job spent in loading/unloading outside, loading inside aircraft compartment, and pushing/pulling carts were 5%, 5%, and 2%, respectively. We used this information to calculate a “physical load index” for each worker, as the average rating of perceived load in both the low back and the shoulders across these tasks, weighted by their occurrence.

### 2.7. General Health and Musculoskeletal Disorders

General health was reported using one question, that is, “In general, how would you rate your health.” The one-year prevalence of low back pain (LBP), shoulder pain (SP), and pain interfering with work (PIW) was retrieved using the Standardized Nordic Questionnaire [18, 19]. The intensity of pain (PINT) during the preceding 12 months was reported on a 10-grade scale from “no pain” to “very very high (almost maximal).”

### 2.8. Data Analysis

Ratings of psychosocial factors in the two domains* Work organization and job content* (five factors) and* Interpersonal relations and leadership *(eight factors) were analyzed both for each factor separately and after combining factor ratings within each of the two domains. In this process, ratings of* role conflict* were reversed. For each of the resulting 15 psychosocial variables, scores were divided into population quartiles and the lower and upper quartile populations were used for comparisons in logistic regression (see below).

Four dichotomized outcomes were addressed in particular, that is, pain interfering with work (PIW) and high pain intensity (PINT) during the preceding year, separately for the low back (LBP) and for the shoulder (SP). Pain interfering with work (PIW) was classified as “yes” or “no.” “High pain intensity” (PINT) was defined as the subject rating 5 or higher on the pain intensity scale for either low back or shoulder. This definition was based on the finding by Andersen et al. [20] in which subjects reporting a pain intensity of 5 or larger are more at risk of eventually suffering long-term sickness absence than those reporting less than 5. A case of “No pain” was registered when the subject reported “no pain” for all body regions.

For all psychosocial factors, Hazard Ratios (HR) with 95% confidence intervals (95% CI) were estimated using Cox proportional hazards regression for the group with upper quartile ratings to have more severe outcomes (PIW and PINT) than the corresponding lower quartile group. HR was adjusted for age, BMI, general health, and physical work load, while we did not adjust for current fatigue. All analyses were done in SAS 9.3 (SAS Institute Inc.).

## 3. Results

Of 806 eligible baggage handlers, 525 (98% males and 2% females) answered the questionnaire, that is, a 65% response rate. General health by age is shown in Table 1. Stratified information was not available for twenty-six subjects.

The one-year prevalence of pain in the low back and shoulders was 70% and 60%, respectively, among those workers answering to the question on pain (missing *n* = 44 for low back and *n* = 53 for shoulders). The different combinations of low back and shoulder pain (Table 2) showed that almost half (45%) of the total population of handlers reported to have both low back and shoulder pain. Among workers reporting low back pain (LBP+/SP+ and LBP+/SP−; *n* = 339), 30% (*n* = 101) reported low back pain only and 70% (*n* = 238) also reported shoulder pain (Table 2). Sixteen percent of workers with shoulder pain reported pain in that region only (LBP−/SP+; *n* = 47), while 84% had also low back pain (LBP+/SP+; *n* = 238). Of the 339 and 285 workers reporting pain in the low back and shoulders, respectively, 328 and 265 proceeded, as intended, to rate whether that pain interfered with work (PIW). Pain intensity (PINT) was rated by more workers than those reporting pain; 506 and 508 workers rated intensity for low back and shoulder pain, respectively. However, 101 and 137 of these workers rated the intensity as 0 or 0.5, that is, effectively an absence of pain. Low back pain more often inhibited work than shoulder pain (Table 2), with 46% (*n* = 156) of those reporting any pain in the low back (*n* = 339) being inhibited by that pain, while pain was inhibiting for only 34% (*n* = 96) of those reporting shoulder pain (*n* = 285).

### 3.1. Psychosocial Work Factors and Means for the Outcome Groups

Mean values for the 13 psychosocial factors and for the combined domains* Work organization and job content* and* Interpersonal relations and leadership* are presented in Table 3 for each of the outcome groups.

In all outcome groups, scores were lowest on* quality of leadership* and* influence *at work and highest on* social community* at work. Overall, values were lower, indicating more dissatisfaction, for the* Work organization* domain than for the* Interpersonal relations* domain.

For all psychosocial factors, baggage handlers with no pain reported better psychosocial working conditions than any of the four pain groups.

### 3.2. Associations between Psychosocial Factors and Pain

Lower ratings in the domain* Interpersonal relations* and, in particular, in the domain* Work organization and job content* were significantly associated with increased occurrence of pain inhibiting work, PIW, both in the low back and in the shoulder region (Table 5). Several separate psychosocial factors in the two domains contributed to this overall association (Table 5). For intense pain, PINT, only* role clarity* showed a significant association with LBP, and this contributed to an overall association with pain for ratings in the domain* Interpersonal relations *(Table 6). For the shoulder, social community at work was associated with intense pain, and* Interpersonal relation* was again the only domain showing a significant association (Table 6). Workers being more dissatisfied with* Interpersonal relations* were, thus, more likely to show both pain interfering with work and intense pain, while workers with a more negative opinion on the* Work organization* reported, to a larger extent, that their work was inhibited by the pain, but not that the pain was intense.

## 4. Discussion

### 4.1. Summary

In this large population of flight baggage handlers, intense low back and shoulder pain and pain interfering with work were both significantly associated with some psychosocial factors at work, as measured by separate scales within the two domains* Work organization and job content* and* Interpersonal relations and leadership* from the COPSOQ II questionnaire [17]. These associations appeared after adjusting for perceived physical load. When scales were combined into the domain* Work organization and job content* we found significant associations with pain inhibiting work, but not with intense pain, while* Interpersonal relations and leadership* were strongly associated with both expressions of pain.* Social community at work* was the strongest single factor explaining intense pain, while pain interfering with work showed a particularly strong association with* possibilities for development* for the low back, and with* social support from colleagues* for the shoulder. The results suggest that psychosocial factors may be important to development and persistence of pain even in occupations characterized by considerable physical loads.

### 4.2. Comparison with Previous Studies

The one-year prevalence of LBP (64%) was similar to or even higher than that found in other occupations requiring heavy manual handling, for example, scaffolders (60%) [21], ambulance workers (60%) [22], and industrial workers (52%) [23]. The one-year prevalence of LBP in the general population varies in the literature between 10% and 56% [24]. The global prevalence of LBP, irrespective of time window, was reported in a systematic review to be 31%, and the prevalence of activity limiting LBP was 17% [25]. Thus, flight baggage handlers appear to have larger low back pain prevalence than the general population. The prevalence of SP among the flight baggage handlers (55%) was similar to previous reports from scaffolders (50%) [21] and ambulance workers (46%) [22]. In the general population, the one-year SP prevalence reported in the literature varies between studies from 5% to 47% [26].

Eighteen percent of the baggage handlers reported shoulder pain interfering with work, which is considerably more than among ambulance workers (7%) [22]. Explanations may be that it is easier to compensate for shoulder pain in ambulance work by engaging in other tasks than lifting and carrying equipment and patients to and from the ambulance and that there may be better possibilities to reschedule work with support from colleagues.

Almost one-third (30%) of the baggage handlers reported low back pain interfering with work, which is somewhat more than the prevalence of activity limitation due to LBP reported by scaffolders (21%) and ambulance workers (23%). Low back pain with perceived disability has been shown to relate significantly to awkward arm postures [21], and a larger occurrence of extreme arm postures in baggage handling, for instance when operating in narrow aircraft compartments, than in ambulance work may explain some of the larger prevalence of LBP. While comparisons of prevalence data between studies must be made with caution because of possible differences in definitions of LBP and SP, the cited studies above of ambulance workers, scaffolders, and industrial workers all used the standardized Nordic Questionnaire to investigate the one-year retrospective prevalence of pain and pain interfering with work. The scaffolder study used a modified expression of pain and pain intensity during the past 12 months, and so quantitative comparisons with our results need to be done with caution.

Higher mean values on the psychosocial rating scales indicate more satisfaction (except for* role conflicts*). Mean scores for the 13 scales varied between 38 and 79 in the entire population of baggage handlers. Factors with the lowest mean scores were* influence at work*,* quality of leadership*, and* predictability* (mean = 37, 38, and 44, resp.), which is somewhat lower than among Danish construction workers (mean = 50, 54, and 54, resp.) (The Danish National Research Center for the Working Environment 2014) [27]. Mean scores were largest for* social community *at work, followed by* role clarity* and* meaning of work* for both baggage handlers and construction workers. These two occupational groups, similar in their rating of psychosocial factors, are both dominated by a male workforce and perform heavy manual handling tasks. The more positive values for* role clarity* and* meaning of work* may be a sign of a vocational pride among the workers.

Several reviews have concluded that lack of social support is a risk factor for pain in low back and upper extremities [11, 13, 28], while others do not claim an association [29]. In our study, limited* social support *was clearly associated with low back and shoulder pain interfering with work. This is consistent with two cross-sectional studies, one of Swedish male ambulance workers [22] where lack of social support was also associated with low back complains and activity limitation and another of the general Canadian working population where low social support was associated with restricted activity due to low back pain [30]. One hypothesis may be that poor social support is a contributing factor to the onset or aggravation of MSDs through a stress response. Lack of support may cause increased muscle tension, as facilitated by stress hormones, which eventually may lead to pain [31]. Social support from colleagues may also be an important factor for coping with pain and staying in the baggage handling job, since it may support, for example, work rotation and task variation. This theory corresponds with Woods [13], who claimed evidence, even if limited, for lack of social support being associated with absenteeism and restricted activity due to MSD. Woods also viewed good social support as an important promoting factor for workers with MSDs to be able to return to work.

### 4.3. Methodological and Theoretical Considerations

This study used a cross-sectional design, and so the observed associations between psychosocial factors and pain can be interpreted to show causal relationships only with great caution. In another cross-sectional study, Davies and Heaney [32] showed that associations between psychosocial work characteristics and pain were stronger when using self-reported pain than when pain was diagnosed in a physical examination. The authors interpreted this to show that psychosocial stressors influence reporting of pain rather than physiologic responses associated with pain and also considered their results to reflect the influence of low back pain on the reporting of poor psychosocial conditions. Thus, cross-sectional relationships between factors at work and pain may, to some extent, be spurious. To this end, it is well known that subjective opinions about the work environment may be influenced by several factors in addition to the health status of the respondent, such as the context of where, how, and when exposure and outcome data were collected. Thus, workers being observant of a relationship between occupational factors and MSDs may both consider exposures to be larger and attribute their possible pain to their work [32]. This possible attribution bias of pain ratings may be particularly pronounced if the workers are required to answer questions while at work, as compared to outside work [33]. Thus, in our study, the administration of questionnaires to be answered during working hours and in the context of a project addressing the work environment may have led to an overestimation of the prevalence and intensity of pain and the extent to which that pain interfered with work.

For reasons of feasibility we assessed pain by self-report, using the NMQ questionnaire, which has been used in a plethora of previous studies since its publication in 1987. Several studies have shown that the pain prevalence obtained when using NMQ is larger than that “confirmed” by clinical examination (e.g., [34]) and thus that specificity might be an issue when using NMQ. However, pain ratings obtained with NMQ have also been shown to have a good predictability with respect to secondary outcomes, such as sickness absence from work [19], and we utilized this property by categorizing workers according to their self-reported pain intensity, using a discrimination limit which is predictive of long-term sickness absence [20].

Many previous studies of psychosocial factors at work have only addressed the standard demand-control-support model or have focused on the Job content questionnaire. We used the validated COPSOQ method, which encompasses several additional dimensions of the psychosocial work environment. COPSOQ is currently a well-established questionnaire for workplace investigations. By using COPSOQ, our study can elucidate even positive aspects of the baggage handlers' working conditions, for example,* social community at work*,* possibilities for development*, and* meaning of work*. In addition to the 13 separate factors included in the two psychosocial domains documented in our study, that is,* Work organization and job content* and* Interpersonal relations and leadership*, we also, to our knowledge for the first time, used these combined domains as independent variables in an analysis for associations with MSDs. Using combined domains renders the study less sensitive to redundant findings on separate factors, caused by the same workers reporting low scores on several factors at the same time.

Mechanisms for causation of MSDs by psychosocial exposures in the presence of physical workloads are not fully clarified. Several theories have been presented [35], one example being the biopsychosocial model, suggesting psychosocial risk factors to exacerbate the effects of physical exposure on the risk for developing MSDs. As an example, according to this model, sociocultural factors such as demand or pressure from colleagues and supervisors and attitudes and behaviors at the workplace may act together with biomechanical and biological factors, such as personal capacities in influencing the work, and modify the risk for MSDs. Mental workloads may even increase muscle tension, which can then lead to biomechanical stress [36, 37] leading to increased muscle metabolites, inflammatory changes, and muscle pain [38]. This influence of mental loads on muscle activation may particularly affect certain low threshold motor units, which may then be prone to develop chronic conditions, according to the “Cinderella” recruitment hypothesis [36].

### 4.4. Generalizability and Implications

Our study is a nationwide investigation of a homogenous occupational group of flight baggage handlers working at the ramp or in sorting, and in spite of some limitations, for instance the somewhat meager response rate, we believe that the results can be generalized to the general population of baggage handlers on a national level and that they may have some portability even to other countries, noting that flight baggage handlers probably have very similar jobs in all major airports worldwide (see below).

Only workers in ramp and sorting areas were included in this study and we therefore believe that the group is reasonably homogeneous with respect to physical workload. However, tasks and work organization may differ, in particular between workers in different airports. We were able to adjust for the general effect of perceived physical load, but data did not permit a nested analysis of associations between psychosocial factors and pain in each of the six airports due to the limited number of workers in some of them (cf.  Table 4). Since the psychosocial conditions appeared to differ between airports, we cannot rule out that some of the observed associations are confounded by airport-specific factors that were not recorded and analyzed, such as variation in physical work load. We chose not to adjust for seniority at work, since it correlates highly with age, which was adjusted for. We did not have access to data on possible confounders describing current acute or systemic disease.

In our data collection, pain could have been a motivating factor for participation, resulting in a study population with an overrepresentation of workers with pain. Other studies have, indeed, shown nonresponders to have less pain than responders [39]. If so, our results may overestimate the prevalence of pain among baggage handlers. In spite of extensive and repeated efforts in retrieving questionnaires from all 806 eligible baggage handlers, we only got a response rate of 65%. While a formal analysis of nonrespondents was not possible, we have the impression that workers on night shifts responded less than day-shift workers, which would limit the representativeness of our results to mainly day shift baggage handling, and also that some team managers were less active in encouraging their team members into participating. It is possible that such nonresponding teams would have a different experience of their psychosocial conditions than teams motivated to participate, but we could, for obvious reasons, not explore that. Differential nonresponding may also have occurred due to workers with a deviating job strain being less inclined to participate [40].

Workers developing MSDs at work are more likely to leave their job, which may lead to an underestimated risk of exposures causing MSDs (the so-called healthy worker effect). However, data provided by the participating handling companies showed that the annual workforce turnover was less than five percent. Thus, despite a considerable prevalence of pain and negative opinions on psychosocial factors, workers stayed at the job. In addition to the harsh conditions in the current labor market in Sweden, the small turn-over may be a result of the valued social community at work.* Social community at work* was scored as the most positive psychosocial factor in our investigation, and the impression of a good social community was confirmed by several informal conversations with the baggage handlers and their union representatives.

Studies have shown that if low back pain becomes chronic, workers may not necessarily be on sick leave [41], but the pain may influence productivity and company costs in a negative way [42]. This agrees with our results of a considerable proportion of workers reporting that their pain inhibited work (PIW). Thus, Heuvel et al. (2010) found psychosocial factors to be more strongly associated with a low performance at work than with sickness absence in a national cross-sectional study of the general Dutch working population [43]. Favorable psychosocial work conditions may therefore have a decisive role in securing that productivity goals are met, such as, for baggage handling, average time spent loading or unloading an aircraft, frequency of departures on time, proportion of baggage being delivered undamaged, and proportion of baggage going to the correct destination.

Airport baggage handling is a world-wide occupation with, to a large extent, similar working conditions, as set out by the standardized construction of airplanes and ramps, and so we believe that our study is of interest even outside Sweden, at least in large- and medium-sized airports. However, we also emphasize that psychosocial conditions may, to a considerable extent, be specific to individual handling companies and that our quantitative results may therefore be difficult to transfer directly to other companies than those investigated. This said, our study revealed associations between psychosocial factors and MSDs, which may be used as a general inspiration for identifying targets for intervention in baggage handling, in addition to possible interventions on the physical workloads.

## 5. Conclusions

We conducted a nationwide study of psychosocial work conditions and musculoskeletal health among baggage handlers within the aviation industry in Sweden. We found the one-year prevalence of low back and shoulder pain to be in parity with other heavy manual occupations. We found significant associations between, on one hand, the psychosocial domains* Work organization and job content* and* Interpersonal relations and leadership*, and, on the other hand, intense pain and pain interfering with work. Thus, while being cross-sectional and therefore only tentatively interpretable in terms of causal relationships, our study suggests that psychosocial factors may be involved in explaining the occurrence of pain in flight baggage handling, in spite of this job also presenting considerable physical loads. Our results also suggest that the psychosocial work environment may be a relevant target for intervention in this occupation.

## Figures and Tables

**Table 1:** General health by age group (*n* with percent of column totals).

General Health	Age (years)				
	All (*n* = 525)	<34 (*n* = 256)	35–49 (*n* = 177)	>50 (*n* = 71)	Age missing (*n* = 21)
Excellent/very good	265 (50)	163 (64)	74 (42)	20 (28)	8 (38)
Good	189 (36)	78 (30)	71 (40)	32 (45)	8 (38)
Somewhat bad/bad	66 (13)	10 (4)	32 (18)	19 (27)	5 (24)
Health rating missing	5 (1)	5 (2)			

**Table 2:** Number of workers (percent of total study population, *n* = 525) reporting any pain (LBP, SP), pain interfering with work (PIW) and high pain intensity (PINT).

Yes (+)	LBP+	LBP+	LBP−	LBP−	Missing answers on pain	LBP	SP	LBP	SP
No (−)	SP+	SP−	SP+	SP−		PIW+	PIW+	PINT+	PINT+
Number of workers *n* (%)	238 (45)	101 (19)	47 (9)	79 (15)	60 (12)	156 (30)	96 (18)	180 (34)	147 (28)

**Table 3:** Mean values of ratings of 13 psychosocial work factors and the combined domains Work organization and job content and Interpersonal relations and leadership in each of the outcome groups; that is, no pain, pain interfering with work (PIW) and high pain intensity (PINT) for low back (LBP) and shoulder (SP).

	All	No pain	LBP PIW	LBP PINT	SP PIW	SP PINT
	*n* = 501	*n* = 79	*n* = 156	*n* = 180	*n* = 96	*n* = 147
	m (SD)	m (SD)	m (SD)	m (SD)	m (SD)	m (SD)
Work organization, job content						
Influence at work	38 (17)	39 (16)	35 (16)	36 (18)	32 (18)	35 (19)
Possibilities for development	46 (17)	48 (17)	43 (16)	45 (15)	42 (16)	45 (15)
Variation	44 (18)	48 (18)	40 (16)	43 (16)	40 (17)	43 (17)
Meaning of work	58 (19)	62 (17)	54 (19)	56 (18)	51 (20)	54 (18)
Commitment to the workplace	47 (29)	53 (22)	40 (18)	43 (20)	39 (20)	42 (19)
Interpersonal relations						
Predictability	44 (20)	47 (21)	40 (18)	41 (19)	38 (20)	40 (19)
Recognition	50 (22)	55 (21)	44 (22)	46 (22)	38 (21)	43 (22)
Role clarity	67 (18)	69 (17)	63 (18)	63 (18)	63 (19)	64 (18)
Role conflicts	46 (17)	44 (16)	50 (16)	49 (16)	53 (16)	50 (16)
Quality of leadership	38 (22)	45 (22)	31 (20)	34 (20)	29 (20)	32 (20)
Social support from colleagues	57 (18)	59 (18)	55 (17)	56 (16)	51 (19)	53 (18)
Social support from supervisors	46 (24)	50 (25)	40 (23)	42 (23)	37 (25)	40 (24)
Social community at work	79 (15)	80 (15)	79 (14)	78 (15)	76 (16)	76 (15)
Work organization	46 (13)	50 (12)	42 (12)	44 (12)	42 (14)	43 (13)
Interpersonal relations	53 (12)	56 (12)	50 (13)	51 (13)	47 (14)	50 (12)

**Table 4:** Mean values of ratings of 13 psychosocial work factors and the combined domains Work organization and job content and Interpersonal relations and leadership in each of the six studied airports.

	All	Airport 1	Airport 2	Airport 3	Airport 4	Airport 5	Airport 6
	*n* = 501	*n* = 330–334	*n* = 100-101	*n* = 34	*n* = 26-27	*n* = 13-14	*n* = 11
	m (SD)	m (SD)	m (SD)	m (SD)	m (SD)	m (SD)	m (SD)
Work organization, job content							
Influence at work	38 (17)	37 (18)	36 (16)	38 (19)	47 (17)	41 (16)	36 (16)
Possibilities for development	46 (17)	44 (17)	49 (16)	49 (15)	55 (17)	53 (11)	52 (11)
Variation	44 (18)	41 (18)	48 (16)	53 (16)	50 (18)	60 (9)	54 (12)
Meaning of work	58 (19)	56 (20)	62 (16)	59 (18)	65 (18)	60 (15)	63 (21)
Commitment to the workplace	47 (29)	45 (17)	54 (19)	48 (21)	52 (22)	51 (18)	45 (17)
Interpersonal relations							
Predictability	44 (20)	45 (19)	42 (21)	41 (19)	57 (17)	28 (15)	22 (15)
Recognition	50 (22)	51 (21)	52 (21)	39 (21)	67 (23)	42 (15)	33 (19)
Role clarity	67 (18)	68 (18)	66 (18)	63 (18)	77 (11)	57 (18)	68 (18)
Role conflicts	46 (17)	46 (17)	46 (17)	51 (12)	42 (15)	54 (14)	48 (6)
Quality of leadership	38 (22)	41 (21)	35 (20)	19 (16)	53 (18)	28 (25)	11 (12)
Social support from colleagues	57 (18)	56 (18)	57 (18)	65 (16)	62 (17)	69 (13)	57 (17)
Social support from supervisors	46 (24)	48 (24)	40 (23)	33 (20)	65 (24)	41 (27)	21 (16)
Social community at work	79 (15)	79 (16)	77 (13)	86 (11)	82 (16)	84 (9)	76 (12)
Work organization	46 (13)	44 (14)	50 (12)	49 (13)	53 (14)	53 (10)	50 (12)
Interpersonal relations	53 (12)	55 (13)	53 (13)	49 (10)	63 (15)	49 (12)	42 (9)

**Table 5:** Hazard ratios (HR) with 95% confidence intervals for associations of psychosocial factors with pain interfering with work (PIW) in the low back (LBP) and shoulder (SP) during the preceding year. All analyses were adjusted for age, BMI, general health and physical work load. Significant HRs marked in boldface.

	LBP PIW		SP PIW	
	HR	95% CI	HR	95% CI
Work organization, job content				
Influence at work	1.60	0.83–3.09	2.12	0.98–4.57
Possibilities for development	**2.86**	**1.32–6.18**	**2.63**	**1.06–6.51**
Variation	**2.31**	**1.08–4.94**	1.33	0.55–3.20
Meaning of work	**2.76**	**1.35–5.61**	2.06	0.86–4.91
Commitment to the workplace	**2.39**	**1.15–4.95**	1.44	0.63–3.29
Interpersonal relations				
Predictability	1.94	0.94–3.98	1.44	0.62–3.37
Recognition	**2.67**	**1.33–5.35**	**2.57**	**1.11–5.95**
Role clarity	1.61	0.76–3.40	1.05	0.45–2.46
Role conflicts	1.25	0.58–2.72	2.08	0.81–5.30
Quality of leadership	1.77	0.82–3.82	1.22	0.51–2.94
Social support from colleagues	**2.48**	**1.16–5.29**	**4.06**	**1.55–10.65**
Social support from supervisors	**2.22**	**1.08–4.58**	1.33	0.60–2.95
Social community at work	0.85	0.42–1.73	1.47	0.67–3.25
Work organization	**3.65**	**1.67–7.99**	**2.68**	**1.09–6.61**
Interpersonal relations	**2.18**	**1.06–4.49**	2.09	0.88–4.96

**Table 6:** Hazard ratios (HR) with 95% CI for associations of psychosocial factors with high intensity pain (PINT) in the low back (LBP) and shoulder (SP) during the preceding year. All analyses were adjusted for age, BMI, general health and physical work load. Significant HRs marked in boldface.

	LBP PINT		SP PINT	
	HR	95% CI	HR	95% CI
Work organization, job content				
Influence at work	1.46	0.86–2.46	1.43	0.83–2.46
Possibilities for development	0.99	0.53–1.84	1.07	0.54–2.11
Variation	0.97	0.53–1.79	0.89	0.47–1.69
Meaning of work	1.02	0.56–1.86	1.57	0.83–2.97
Commitment to the workplace	1.17	0.65–2.10	1.55	0.84–2.88
Interpersonal relations				
Predictability	1.59	0.88–2.88	1.70	0.91–3.17
Recognition	1.58	0.88–2.48	1.83	0.98–3.41
Role clarity	**2.07**	**1.08–3.95**	1.81	0.94–3.50
Role conflicts	1.17	0.61–2.24	1.76	0.88–3.53
Quality of leadership	1.76	0.91–3.42	0.98	0.50–1.95
Social support from colleagues	1.08	0.57–2.03	1.79	0.92–3.49
Social support from supervisors	1.24	0.67–2.28	0.96	0.51–1.80
Social community at work	1.61	0.89–2.93	**2.21**	**1.18–4.13**
Work organization	1.22	0.66–2.24	1.30	0.69–2.44
Interpersonal relations	**1.95**	**1.05–3.65**	**2.11**	**1.08–4.12**

## Appendix

### A. Questions on Psychosocial Factors as Appearing in the Second, Updated Version of the Standardized COPSOQ Questionnaire [17]

#### A.1. Work Organization and Job Contents (Five Scales)

*Influence at Work (Four Questions).* Do you have a large degree of influence concerning your work? Do you have a say in choosing who you work with? Can you influence the amount of work assigned to you? Do you have any influence on what you do at work?

*Possibilities for Development (Four Questions).* Does your work require you to take the initiative? Do you have the possibility of learning new things through your work? Can you use your skills or expertise in your work? Does your work give you the opportunity to develop your skills?

*Meaning of Work (Three Questions).* Is your work meaningful? Do you feel that the work you do is important? Do you feel motivated and involved in your work?

*Variation (Two Questions).* Is your work varied? Do you have to do the same thing over and over again?

*Commitment to the Workplace (Four Questions).* Do you enjoy telling others about your place of work? Do you feel that your place of work is of great importance to you? Would you recommend a good friend to apply for a position at your workplace? How often do you consider looking for work elsewhere?

#### A.2. Interpersonal Relations and Leadership (Eight Scales)

*Predictability (Two Questions).* At your place of work, are you informed well in advance concerning, for example, important decisions, changes, or plans for the future? Do you receive all the information you need in order to do your work well?

*Recognition (Three Questions).* Is your work recognized and appreciated by the management? Does the management at your workplace respect you? Are you treated fairly at your workplace?

*Role Clarity (Three Questions).* Does your work have clear objectives? Do you know exactly which areas are your responsibility? Do you know exactly what is expected of you at work?

*Role Conflicts (Four Questions).* Do you do things at work, which are accepted by some people but not by others? Are contradictory demands placed on you at work? Do you sometimes have to do things which ought to have been done in a different way? Do you sometimes have to do things which seem to be unnecessary?

*Quality of Leadership (Four Questions).* To what extent would you say that your immediate superior: makes sure that the individual member of staff has good development opportunities? gives high priority to job satisfaction? is good at work planning? is good at solving conflicts?

*Social Support from Colleagues (Three Questions).* How often do you get help and support from your colleagues? How often are your colleagues willing to listen to your problems at work? How often do your colleagues talk with you about how well you carry out your work?

*Social Support from Supervisors (Three Questions).* How often is your nearest superior willing to listen to your problems at work? How often do you get help and support from your nearest superior? How often does your nearest superior talk with you about how well you carry out your work?

*Social Community at Work (Three Questions).* Is there a good atmosphere between you and your colleagues? Is there good cooperation between the colleagues at work? Do you feel part of a community at your place of work?

## Abbreviations

ULD: Unit load device
MSD: Musculoskeletal disorders
LBP: Low back pain
TYA: The Vocational Training and Working Environment Council
COPSOQ: Copenhagen Psychosocial Questionnaire
SP: Shoulder pain
PIW: Pain interfering with work
PINT: Pain of high intensity.
